# The efficacy of oligonucleotide-based gene therapeutics in gene silencing

**DOI:** 10.7150/thno.121775

**Published:** 2026-01-01

**Authors:** Christina Patra, Zain Hussein, Veronika D. Ace, Elena V. Misnik, Daria S. Rybalko, Adeliia A. Salimova, Daria S. Ereshko, Mikhail V. Dubovichenko, Moustapha A.Y. Nour, Valeriia S. Drozd, Dmitry M. Kolpashchikov

**Affiliations:** 1 Laboratory of DNA-nanosensor diagnostics, ITMO University, Saint-Petersburg, 191002, Russian Federation.; 2 Institute of Gene Biology, Russian Academy of Sciences, Moscow, Russian Federation.; 3 Pediatric Research and Clinical Center for Infectious Diseases, Saint Petersburg, Russian Federation.; 4 Department of Biomedical Sciences, University of Padova, 35131 Padova, Italy.; 5 Infochemistry Scientific Center, ITMO University, Saint Petersburg, 191002, Russian Federation.; 6 Chemistry Department, University of Central Florida Orlando, FL 32816-2366, USA.; 7 Burnett School of Biomedical Sciences, University of Central Florida, Orlando, FL 32816, USA.

**Keywords:** gene silencing, therapeutic oligonucleotides, efficacy, clinical relevance, cancer, antisense oligonucleotides, siRNA, miRNA, ribozymes, deoxyribozymes, CRISPR/Cas

## Abstract

Oligonucleotide-based gene therapeutics (OGTs) have emerged as a promising strategy for treating a variety of diseases, offering a tool for gene modulation at the mRNA level. Despite significant progress in OGTs development, their efficacy in both experimental and clinical settings has often fallen short of expectations. Current estimates suggest that less than 1% of transfected OGTs are released into the cytosol, significantly limiting the interaction with target RNA. Moreover, data suggests that only about 2% of the tested siRNAs achieve the expected 70% target gene knockdown *in vitro*. Clinically approved OGTs appear to be effective only against genetic disorders that lack effective alternative treatment, and even in these cases their therapeutic contribution remains marginal. Notably, the majority of approved OGTs, as well as those currently in clinical trials, are antisense oligonucleotides (ASOs) despite cell culture data showing that small interfering RNAs (siRNAs) exhibit greater potency. The delayed commercialization of siRNAs, despite high research interest, may be attributed to passenger stand-dependent off target effect and the immaturity of their design and modification strategies. This review critically evaluates the factors influencing therapeutic efficacy of OGTs and highlights the persistent gap between theoretical promise and clinical reality.

## 1. Introduction

Gene therapy has been regarded as a transformative approach for treating hereditary or acquired diseases, including cancer. Its core principle is the introduction of genetic material into a patient's cells to replace defective genes with healthy counterparts, aiming to achieve therapeutic benefits 1,2. Among various strategies, gene silencing, a technique which targets and suppresses disease promoting genes at the mRNA level, has emerged as a more refined approach to gene therapy. Within this context, oligonucleotide-based gene therapeutics (OGTs) have received a significant attention as potential game-changers 3.

The principles underlying OGTs rely on complementary base pairing between the therapeutic oligonucleotide (ON) and the target mRNA, leading to either mRNA degradation or steric hindrance of the translation machinery. OGTs include RNAi inducing agents, (e.g. siRNA), antisense oligonucleotides (ASOs), DNAzymes (Dzs) and RNAzymes (Rzs) as well as Crispr/Cas13-based systems. The therapeutic potential of an OGT is characterized by its efficacy in silencing the targeted genes and its specificity, which is also reflected by the extent of off-target effects. While a recent comprehensive review has addressed the specificity of OGT 4, this work focuses on the analysis of OGT efficacy.

In this review, OGT efficacy is defined as the degree to which an OGT can reduce the expression of a targeted gene. Efficiency, on the other hand, refers to the practical performance of OGT under specific conditions, including the amount of OGT required to achieve a biological effect, the speed of its action, and the conditions under which it operates. Thus, while efficacy measures the outcome, efficiency is about optimizing the conditions and resources to achieve this outcome. Both terms are used according to these definitions in this review.

Current research endeavors are focusing on refining the OGT design and delivery systems 5-7. Although productive internalization and release are critical for achieving high efficiency, these aspects are beyond the scope of this review, as the diverse array of delivery systems has been extensively reviewed 8,9. Instead, this review focuses on evaluating current knowledge on OGTs through the lens of the efficacy demonstrated in experimental and clinical contexts. We evaluate the contribution of various factors into OGT efficacy and examine how closely experimental efficacy aligns with theoretical expectations.

## 2. Mechanisms of action and their contribution to OGT efficacy

### 2.1 Antisense Oligonucleotides

**Figure 1:**
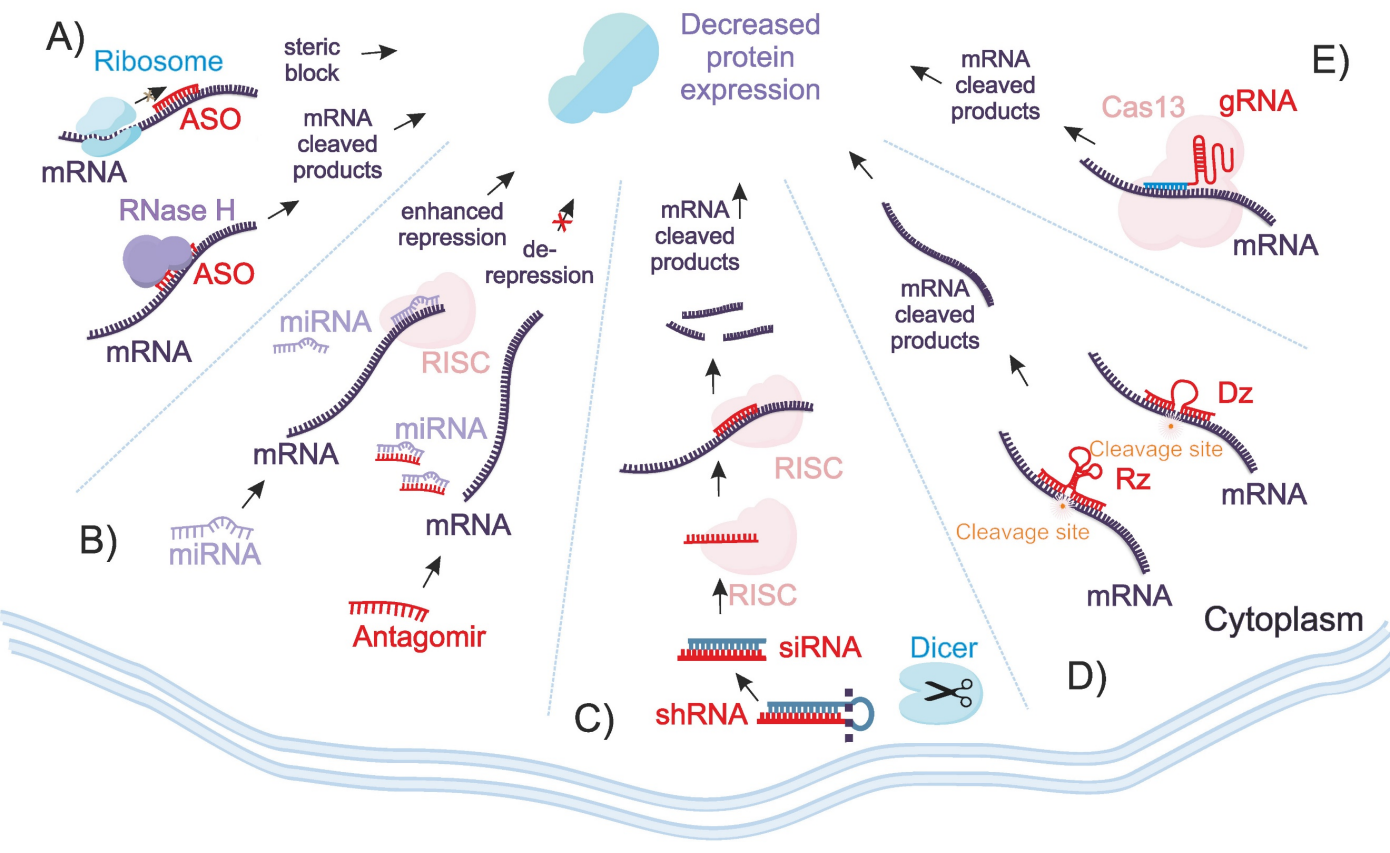
Oligonucleotide-mediated regulation of gene expression. A) ASOs bind to complementary mRNA sequences, leading to either RNase H-mediated degradation of the mRNA or steric inhibition for translation or splicing. B) the RISC complex associated with endogenous miRNA degrades imperfectly complementary mRNA targets, while exogenous antagomirs bind to miRNAs to inhibit their functions, thereby preventing suppression of their targets. C) the RISC complex guided by siRNAs strand degrades complementary mRNA target. Unlike ASOs, siRNAs require enzymes for their activity. D) Dzs and Rzs OGTs are ssDNA and ssRNA molecules with RNA cleaving activity. Unlike RNAi, CRISPR/Cas13, and ASOs, their activity is protein enzyme independent. E) The CRISPR/Cas13 system targets mRNA using crRNAs.

ASOs are short, single-stranded DNA (ssDNA) or their analogs designed to hybridize with a specific mRNA sequence (Figure 1A). Their mechanism of ASO action involves binding to the target mRNA, which can lead to two outcomes - (1) RNase H mediated mRNA degradation or (2) steric blockage. In the first mechanism, the mRNA component of the ASO/RNA heteroduplexes is degraded by RNase H1 in the cytoplasm or by RNase H2 in the nucleus 10,11. In steric blockage either induce translational arrest by hindering interactions with ribosomal subunits or modulates splicing by influencing exon skipping or inclusion 10.

### 2.2 RNA Interference (RNAi) Agents

In 1998, Fire and Mello revealed the mechanism of RNA interference (RNAi) for the regulation of gene expression in the nematode Caenorhabditis elegans 12. RNAi agents, including small interfering RNAs (siRNAs), short hairpin RNAs shRNAs and microRNAs (miRs), induce degradation of the specific mRNA target by harnessing the RNA-induced silencing complex (RISC) (Figure 1B-C). Guided by a short ssRNA template, RISC recognizes the complementary mRNA target via Watson-Crick base pairing, leading to mRNA degradation and inhibition of protein synthesis 13. Unlike ASO, can induce silencing without involvement of endogenous enzymes, RNAi agents differ are double-stranded RNAi agents that require RISC for their activity 14.

### 2.3 DNAzymes (Dzs) and RNAzymes (RZs)

DZs and RZs are synthetic single stranded nucleic acids that possess catalytic activity 15-17. Both RZs and DZs can be engineered to cleave particular mRNA sequences 18,19. Both can be designed to recognize particular mRNA sequences with high specificity due to their short RNA binding 20,21. However, their catalytic efficiency is often limited by the relatively low binding affinity of these short recognition arms 22. Unlike RNAi, CRISPR/Cas agents and ASOs, DZs and RZs activity is completely protein enzyme-independent (Figure 1D).

### 2.4 CRISPR/Cas13

CRISPR/Cas13 is a member of the CRISPR/Cas family that has gained significant attention due to the unique ability to target RNA rather than DNA, thereby enabling gene manipulation at the transcriptional level 23. Similar to other CRISPR/Cas systems, Cas13 proteins are guided by CRISPR RNAs (crRNAs) to complementary sequences of targeted RNAs 24,25 (Figure 1E). There are currently four main subtypes of Cas13 - Cas13a, Cas13b, Cas13c, and Cas13d 23. Upon binding of the crRNA to the target RNA via Watson-Crick base pairing, Cas13 undergoes a conformational change that activates the catalytic site followed by cleavage of the target RNA 26. Unlike other OGTs, CRISPR/Cas13 relies on the expression or co-delivery of the bacterial Cas 13 protein along with the crRNA, introducing an additional challenge in comparison with all other OGT.

Among all OGTs, ASOs and siRNAs remain at the forefront of successful clinical applications. In cell culture, siRNAs often demonstrate greater efficiency at lower concentrations compared to ASOs 27. One major contributing factor is the RISC, which is more stable in cytoplasm, and processive 28, compared to the transient and less processive RNase H/ASO/mRNA complex 29. In a direct comparison between siRNA and ASO efficiency against Influenza A viral RNA, Piasecka *et al.* showed that 8 nM siRNA achieved >84% decrease in viral RNA copies while ASO at the same concentration showed negligible activity 30. Even at a much higher concentration of 750 nM ASO achieved only about 27% decrease in viral RNA level. Interestingly, despite the superior potency of siRNAs in vitro, ASOs have shown greater clinical success, with more approvals from Food and Drug Administration (FDA) and the European Medicines Agency (EMA) over the last two decades (Table 1). In contrast, siRNAs only entered the market in the last seven years 31.

On the other hand, the therapeutic application of miRs is limited due to challenges with off-target effects and lack of specificity that stems directly from their natural function to regulate activity of multiple mRNA targets 32. Similarly, CRISPR/Cas13 faces specificity challenges due to the collateral RNA cleavage, also known as collateral damage, which occurs due to the physical separation between the crRNA/RNA complex and the catalytic site of Cas13 33-35. Dzs and Rzs, while promising *in vitro*, have not yet achieved clinical translation mainly due to the stability issues, low affinity to the folded mRNA as discussed below and, possibly, due to the low intracellular concentration of Mg^2+^, a co-enzyme required for their catalytic activity. dependence.

## 3. The use of OGT-based drugs is limited by their low efficacy

### 3.1 The clinical efficacy of antiviral OGTs cannot yet compete with that of established treatments

While OGTs against infectious diseases have shown promising results in experimental research, their progress through clinical trials has been challenging 36. Fomiversen, developed against Cytomegalovirus (CMV) retinitis, was the first antisense-RNA agent to gain FDA approval in 1998 and remains the only antiviral OGT to have passed clinical trials and reached the market. However, it was discontinued less than a decade later due to the low demand and market competition against the new and highly efficient antiretroviral treatments of the time 37.

Since then, several OGTs have entered clinical trials against viruses including Respiratory Syncytial Virus (RSV), Hepatitis B Virus (HBV), Ebola Virus and Human Immunodeficiency Virus (HIV). Among those, VIR-2218 and JNJ-3989 are currently in ongoing phase II trials for chronic HBV include. VIR-2218 is a N-Acetylgalactosamine (GalNAc) conjugated version of ALN-HBV - an earlier siRNA agent from the same company. VIR-2218 demonstrated improved safety: no elevation in liver inflammation markers was observed, compared to 28% elevation in those treated with ALN-HBV. The VIR-2218 efficiency was evident by dose-dependent reduction in Hepatitis B surface antigen (HbsAg) 38. Treatment of HBV patients with JNJ-3989 provided sustained HBsAg reduction for up to 336 days after the last dose, although complete HBsAg clearance remained rare as demonstrated in the recent phase IIb study 39. RG6346, a new agent against HBV in phase I trials, has also showed favorable safety and pharmacodynamic profile with reductions in HBV protein levels, although efficacy data are not yet available 40.

Other OGTs in phase II trials such as ALN-RSV01 against respiratory syncytial virus (RSV) and TKM-130803 against Ebola virus were discontinued due to the failure in meeting the target suppression efficacy and, in the case of Ebola, the lack of improvement in patient survival 36. Interestingly, OGTs against HIV have been in phase I trials since their initiation in 2007, largely due to the complexity of HIV infection dynamic and lack of comprehensive pre-clinical studies 36.

Overall, the stagnation of antiviral OGT development can be attributed to persistent concerns regarding efficacy and safety. Moreover, the availability of established antiviral therapies presents a significant barrier to adopting OGTs. When existing treatments are effective, there may be less incentive for healthcare providers to switch to new therapies that have not yet demonstrated clear clinical superiority.

### 3.2 In mitigating genetic disorders, clinical efficacy is satisfactory due to the low competition from alternative treatments

For many genetic disorders, existing therapies are limited to symptom management and supportive care. The 16 OGTs approved for genetic disorders (Table 1) partially address the scarcity of disease-modifying treatment options. However, their perceived success is influenced by the lack of alternative effective treatments, making OGT the only treatment available. For example, Mipomersen, the first ASO approved for treating Homozygous familial hypercholesterolemia, was withdrawn due to market competition. Although Mipomersen could decrease low density lipoprotein cholesterol (LDL-C) by 28-36% and apolipoprotein B (ApoB) by 36-38%, it also carried a significant risk of hepatotoxicity 37,41. Safer and more efficient pharmacological alternatives, such as monoclonal antibody-based PCSK9 inhibitors and statins that reduce LDL-C more than 50%, were preferred. Similarly, Volanesorsen for Familial Chylomicronemia Syndrome (FCS) faced FDA rejection due to safety concerns 42 and Tominersen for Huntington's Disease was discontinued due to lack of efficacy 43.

Furthermore, even the efficacy of FDA approved drugs, like Eteplirsen for some types of Duchenne muscular dystrophy, is under question. Patients receiving Eteplirsen weekly showed an increase in muscle dystrophin levels by only ~0.44% ± 0.43% of that of healthy individuals, up from a baseline of ~0.16% ± 0.12% with the median increase just 0.1% after 48 weeks. The EMA has not approved Eteplirsen, citing insufficient evidence of its efficacy 44. This example highlights both differences in regulatory standards and the broader challenges of OGT in providing meaningful clinical outcomes.

A notable example of successful clinical OGT applications are the FDA and EMA approved gene therapies for hereditary transthyretin-mediated amyloidosis (hATTR). hATTR is caused by the deposit of both mutant and wild-type transthyretin (TTR) variants mainly in the nervous system, causing severe multisystem neurological manifestations. Traditional treatment strategies for hATTR include TTR stabilizers, liver transplantation, as well as neuropathy and cardiomyopathy management. All four approved OGTs, Patisiran 45, Vutrisiran 46, Inotersen 47, and Eplontersen (ASO currently under review) 48, target a specific genetically conserved region in the 3' untranslated region (3'-UTR) of all TTR isoforms. Each of these agents demonstrates the same significant efficacy of ~80-85% TTR knockdown. Notably, siRNA-based treatment requires lower and less frequent dosing compared to ASO (Table 1), supporting earlier observation that siRNAs are more potent than ASOs.

However, some siRNAs have encountered setbacks in clinical trials. One example is Revusiran targeting hATTR, which was withdrawn after the randomized, double-blind, placebo-controlled Phase III trial 49. The trial was terminated in 2016 due to higher mortality observed in the treatment group compared with placebo 50. Another failed RNAi agent is Fitusiran. It was developed to treat hemophilia A and B by targeting antithrombin to increase thrombin generation, thus promoting clot formation. However, during clinical trials, patients experienced serious thrombotic events, leading to a temporary suspension of the trials in 2017 and again in 2021. Although, trials have since resumed and completed, comprehensive safety and efficacy data have not been fully evaluated yet 51.

While not classified as OGT, Imetelstat is worth mentioning here due to its approval by the FDA in June 2024 and EMA in March 2025. Imetelstat is a 13-mer DNA oligonucleotide that acts as a first-in-class telomerase inhibitor, functioning not through gene silencing but via hybridization-dependent inhibition of enzyme active site. Clinical data suggest that Imetelstat restores normal haematopoiesis in patients with low-to-intermediate risk myelodysplastic syndromes and transfusion-dependent anaemia 52. Other emerging modalities such as circular RNA and tRNA-derived fragments display significant regulatory properties that could be used for therapeutic purposes. However, their development is at an early stage with efficacy data being limited to date 53-55.

### 3.3 In cancer, clinical efficacy of OGTs is weak and relevant only as complementary treatment

The targets selected for cancer gene therapy encompass a wide range of molecular and genetic factors associated with cancer initiation, progression, and treatment response. These targets include oncomarkers such as dysregulated angiogenic factors, tumor suppressor genes, drug resistance genes like MDR1, and proteins such as survivin and VEGF, all of which underscore the potential of gene therapy to address diverse aspects of cancer biology. However, most currently utilized targets demonstrate insufficient efficacy in cancer cell elimination, poor treatment specificity, and are considered unsuitable as a monotherapy against cancer 56,57. As of 2022, approximately 70 anticancer ASO and 20 of siRNA entered clinical trials. Of these, only two ASOs reached phase III 58, and none have received FDA approval to date.

Bcl-2 mRNA remains one of the most studied targets in anticancer OGT development. For example, BP1002 and PNT2258 entered phase I, while Oblimersen (G3139) passed phase III trials. Bcl-2 silencing by Oblimersen aimed to restore cancer cell sensitivity to chemotherapy. However, when combined with chemotherapeutic agents such as cisplatin and 5-fluorouracil, it achieved only slight improvement of 43% in overall survival compared to 40% in the control group 59. Another study reported that AZD4785, which targeted mutated KRAS mRNA and successfully completed phase I clinical trials, but no updates have been published since 2017 60. Similarly, VEGF-targeting ASOs achieved therapeutic effect in combination with chemotherapeutics pemetrexed and cisplatin 61. Phase I/II trials for VEGF-ASO were completed in 2011 without any further updates.

Except for approaches that seem to sensitize cancer cells to chemotherapeutics 62 other combination strategies under investigation include OGTs engineered to improve radiotherapy sensitivity 63, and dendritic cell immunotherapy response 64, although these approaches have not been tested in clinic yet. Collectively, these data support the view that monotherapy targeting traditional mRNA may not be sufficient to address the complexities of cancer thus highlighting the necessity of finding alternative approaches.

On the other hand, protein inhibitors for the same targets have been effective both as monotherapy and in combination with immunotherapy even against targets that were previously considered “undruggable”. The most popular Bcl-2 small molecule inhibitor, Venetoclax, was FDA approved in 2016 for the treatment of chronic and acute myeloid leukemia 65. These results indicate that OGTs targeting Bcl-2 currently cannot compete with the small molecule inhibitors in terms of efficacy. Similarly, in 2022, the FDA approved Krazati, a KRAS inhibitor, for patients with non-small cell lung cancer 66.

**Figure 3:**
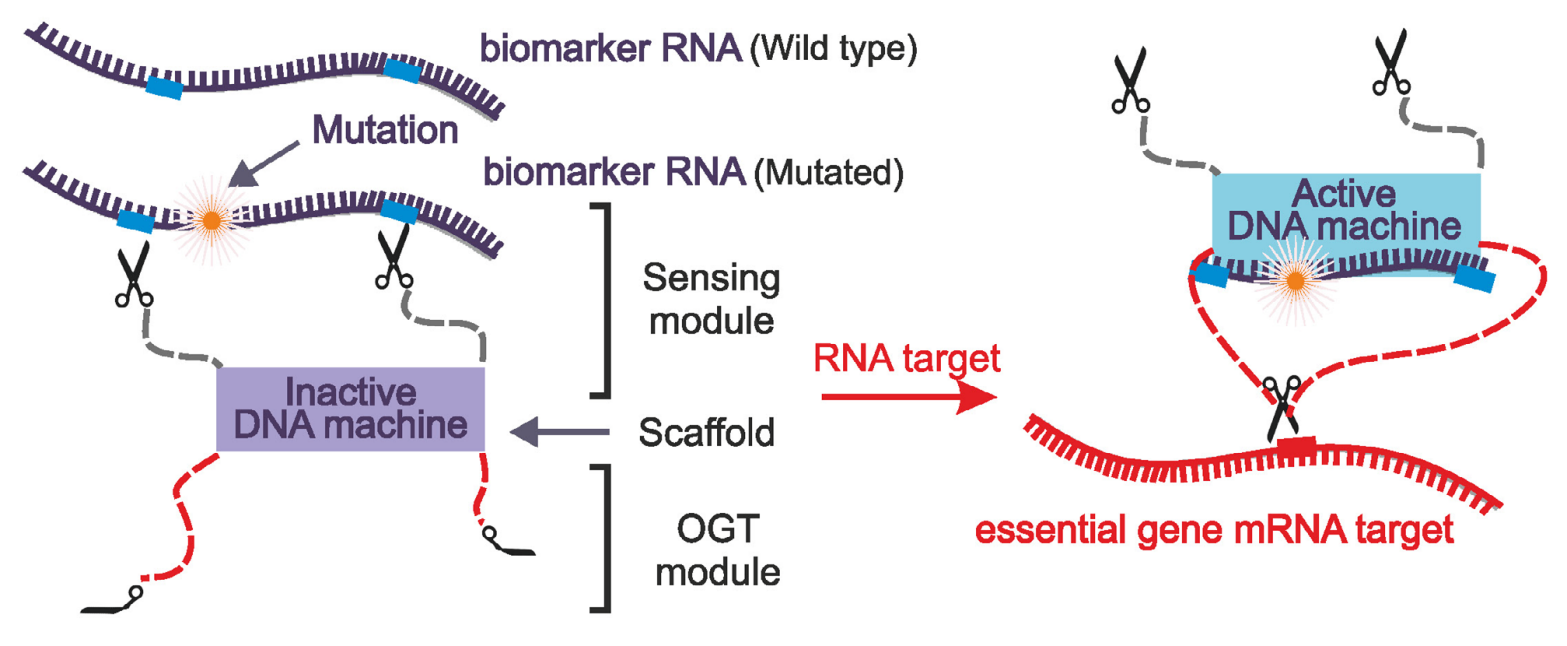
Conditionally activated OGT for the knockdown of vital genes 67,76. The inactive DNA machine recognizes the mutated biomarker's RNA and cleaves it at predetermined sites. The cleavage product is then used to activate the DNA machine which acts as an OGT and silences the mRNA of the target essential gene, ultimately inducing apoptosis in cancer cells.

We believe that the idea of targeting cancer-related genes is fundamentally defective, as it merely suppresses malignant traits of the cancer cells rather than irradicates cancer cells, which is the ultimate therapeutic goal (Table 2) 67. A more promising strategy could be the conditional activation of programmable agents triggered by cancer-specific genes, followed by downregulation of the vital genes that triggers cancer cell death. Several research groups have taken advantage of the programmability of nucleic acids to develop nucleic acid-based nanostructures that release the OGTs upon recognition of cancer markers. Examples include siRNA probes that are converted into Dicer substrate siRNA by an RNA trigger 68, reconfigurable nucleic acid nanoparticles that elicit a therapeutic siRNA response in the presence of mutated KRAS 69, RNA/DNA hybrids that re-assemble into active siRNA in the presence of their cognate hybrids 70, endogenous miRNA-triggered DNA nanostructures for the release of multiple, multifunctional siRNAs 71,72, and miRNA-triggered ASO release 73. Our group has proposed targeting mRNA of vital genes exclusively in the presence of cancer-related mRNAs using marker-dependent Dz and ASO agents 67,74. They include binary ASO that is active only in presence of KRAS RNA 75, binary Dz nanomachines 76, Dzs and ASOs that are activate at high but not low concentrations of miRs 77-79, and Dz-based logic gates that operate based on miRNA expression patterns 80. The principle of DNA nanomachines for the conditional activation of OGTs is shown in Figure 3. The 'Cut' function of these nanomachines recognizes cancer-related mutations with high specificity and cleaves mRNA at two sites to release the cancer marker fragment. This fragment is retained by the DNA nanomachine and serves as an activator either the Dz or ASO function, which suppresses the targeted vital gene, ultimately triggering apoptosis in cancer cells.

Considering the variability in vital gene expression across different tissues, target selection for such programmable systems should be tailored to specific cell type, taking into consideration environmental stressors including hormonal status 81,82. Beyond selecting cancer-related activator genes chosen solely based on the individual cancer type and its dysregulations, this approach aligns with the principles of personalized medicine. The use of programmable OGTs capable of implementing the principles of Boolean logic could also offer a solution to cancer cell heterogeneity by enabling processing complex gene expression patterns and incorporating the synthetic lethality approaches.

## 4. Factors contributing to low OGTs' efficiency

### 4.1 Both the tissue and the cell specific delivery are not efficient

One of the major limitations of the clinical success of OGT is the efficient and tissue-specific delivery of these agents. Naked ONs face a short half-life when in the bloodstream owing to nuclease dependent rapid degradation 83. Advances in chemical modifications and delivery platforms have partially improved this problem, optimizing the biodistribution of OGTs. Biodistribution, however, remains a critical factor influencing therapeutic efficacy. For example, after systemic administration, drugs often accumulate in the liver due to interactions with lipid transport proteins, reducing their chances to reach target tissues. Key physicochemical properties such as size, shape, and charge significantly influence biodistribution. For example, overly small or negatively charged nanoparticles have higher clearance rates 84,85. Since systemic administration often results in poor extrahepatic targeting, alternative delivery routes can enhance tissue accumulation and therapeutic efficacy 86-88. Local administration is generally preferred, as it requires lower doses and is less invasive 89.

Lipid-based delivery systems are currently among the most effective non-viral methods for *in vivo* nucleic acid delivery, with several products approved for clinical use (Table 1), while many candidates remain in trials. Although lipid-based systems exhibit passive targeting capabilities, their lipid composition influences the protein corona on their surface, affecting tissue preference 90. Targeting efficiency can vary from approximately 5% to 90% depending on cell type 90, yet these systems still predominantly accumulate in the liver 91 due to association with ApoE and low density lipoprotein receptor (LDLR)-mediated endocytosis 92. While this property favors hepatocyte targeting, it limits efficacy in other tissues and increases metabolic degradation in the liver 90. Enhancing these systems with cell-specific penetrating proteins (CPPs) or antibodies, or direct bioconjugation with OGTs may help address these challenges 93.

Direct bioconjugation of OGT with lipids, cell penetrating peptides (CPPs), aptamers, antibodies, and sugars, enhances delivery by improving the specificity, cellular recognition, internalization and overall efficiency. CPPs have shown efficacy in targeting tissues such as skeletal muscle 94, heart 95, and the central nervous system 96, although transfection efficiencies varies depending on the cell type 97,98.

Bioconjugation with lipid moieties, such as cholesterol, enhances delivery by promoting endosomal escape than CPPs, primarily through facilitating membrane fusion and endosomal destabilization 93,99-102. The endocytosis of cholesterol-OGTs is mediated by scavenger receptor type B1 (SCARB1, SR-B1) or LDLR, for HDL and LDL particles, respectively 103, leveraging the body's endogenous lipid transport and uptake system *in vivo* 104, though this approach favors hepatic accumulation over others tissue 105. GalNAc conjugates, several of which are clinically approved (Table 1), are particularly effective for liver-targeted delivery. These conjugates bind to the asialoglycoprotein receptor (ASGR) for rapid internalization via clathrin-dependent endocytosis 106. Experimentally, modifications such as 5′-(E)vinylphosphonate 107 and β-cyclodextrins 108 have been shown to enhance potency and stability of GalNAc-conjugated OGTs by 5-10 fold. Triantennary GalNAc, featuring three GalNAc sugars branching from a central core, significantly improves binding and efficacy 106,109.

Exosomes have also gained attention over the past decade due to their natural transport capabilities, long circulation time, and biocompatibility, making them promising vehicles for OGTs 110,111. These lipid bilayer-encapsulated vesicles naturally facilitate intercellular communication and can traverse biological membranes 112, while evading phagocytosis and enhancing bioavailability 113,114. Human exosomes are particularly promising due to their low immunogenicity and compatibility with RNA silencing applications 115. They can be derived from patients to minimize immune responses 116 and exhibit natural tropism toward their parent cells, with potential for engineering enhanced targeting capabilities 117. Exosomes release cargo through surface receptor interactions, fusion with the plasma membrane, or endocytosis via the endolysosomal pathway 118. They effectively protect cargo from lysosomal degradation and facilitate release, demonstrating strong target gene silencing 119,120 and tumor size reduction 121,122. Ongoing research focuses on optimizing exosome properties for therapeutic applications 123-125 with emphasis on improving cargo loading and interactions with sorting proteins 126.

Although non-viral methods generally exhibit lower transfection efficiencies compared to viral vectors, progress is being made in optimizing their formulations 127. Specifically, transfection efficiencies up to ~10% have been reported for the most promising lipid-based systems 128, although this depends on various factors. The cell cycle phase plays a crucial role in intracellular delivery with mitotic with mitotic cells showing higher internalization rates 129,130. The chemistry of both the delivery vehicle and the OGT itself also significantly impacts the productive uptake in different cell and tissue types 105. These parameters complicate platform evaluation and raise concerns about reproducibility across different biological systems.

### 4.2 OGTs' concentrations affect the efficiency of cellular uptake

The concentration of OGTs is a critical determinant of their efficacy and safety in gene therapy applications. While higher concentrations may have higher efficacy, both *in vitro* 131 and *in vivo* 132, they can also induce stronger immune responses 132,133 and cellular toxicity 134, complicating data interpretation and reducing the clinical significance of such treatments. Conversely, lower concentrations may minimize toxicity and adverse effects but generally are expected to reduce gene silencing efficacy. Traditionally, in siRNA studies, concentrations as low as 1-10 nM are considered sufficient for effective target gene silencing in cell culture models 135. Concentrations, such as 50 nM or 100 nM, are often considered high and may induce off-target effects and cellular toxicity without significantly improving silencing 136. In ASO studies, concentrations between 10 - 100 nM are often found to be optimal, while concentrations 100 - 1 µM are typically categorized as high.

The concentration of OGTs directly affects the loading efficiency onto delivery vehicles and their ability to penetrate cell membranes and reach intracellular compartments. For example, Liu Yang *et al.* reported that the gene silencing efficiency improved from 10.9% to 79.5% as the nitrogen in lipids to phosphate in ASO ratio (N/P) increased from 2.5 to 15, highlighting that effective loading enhances cellular uptake and subsequent gene silencing. At the highest tested ASOs concentrations of 7.5, 15 and 30 nM, more than 50% target silencing was achieved, while cell viability remained above 75%. This data indicates that efficient liposome loading correlates with silencing efficacy, but also suggests a threshold beyond which further increases in concentration do not yield proportional improvement in gene silencing 137. Similarly, Xie *et al.* reported that siRNA delivery via transferrin-polyethyleneimine (Tf-PEI) resulted in effective gene silencing in activated T cells. Tf-PEI polyplexes fully condensed 50 pmol of siRNA at an N/P of 7.5 but showed more productive internalization at higher N/P of 10, 15 and 20, with N/P of 15 reaching approximately 60% target gene downregulation 138. These data underscore the importance of understanding the dose-response relationship and the biological mechanisms to advance the development of effective gene therapies.

### 4.3 The endosomal escape efficiency is low

In addition to tissue specificity, the efficient internalization and release of OGTs into cell cytoplasm is another critical factor of therapeutic success. Either naked or encapsulated in delivery vehicles, OGTs are typically internalized via endocytosis 139. Endosomal escape is the process by which the endosomal cargo exits the endosome to reach its target site within intracellular compartments. However, it has been estimated that approximately 99% of OGTs remain entrapped within endosomes, hindering their cytosolic delivery and subsequently reducing treatment efficacy 140,141. This entrapment may result in the two possible outcomes; i) natural maturation of early endosomes to late endosomes and lysosomes, where the cargo is eventually digested; or ii) the formation of a poorly defined so-called “depot endosome” in which the cargo remains intact for extended periods.

**Figure 4:**
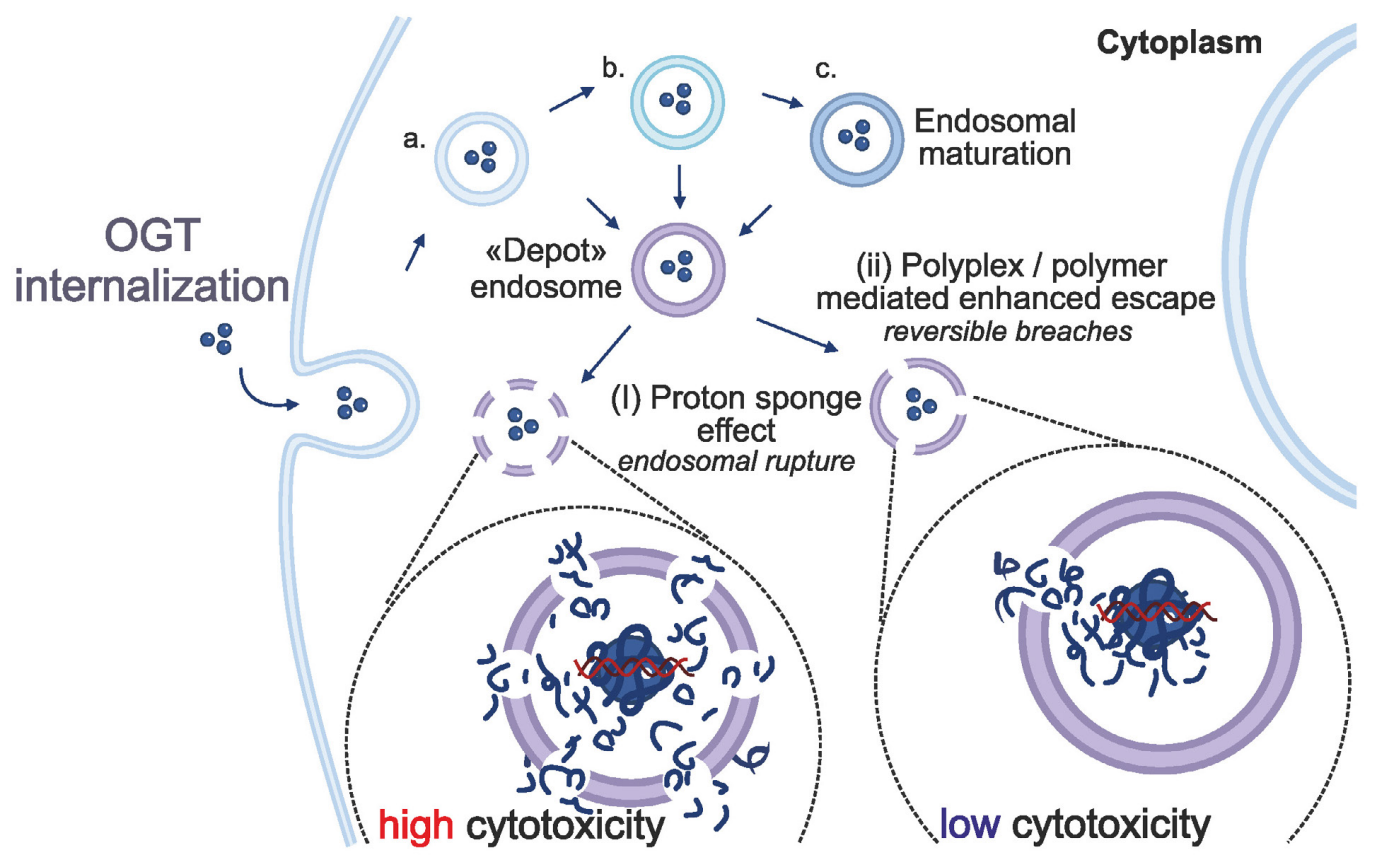
Possible fates of internalized OGTs. OGTs are not capable of diffusing through the cell membrane; thus they are taken up through endocytosis. Instead of undergoing maturation, “depot” endosomes can be formed, and release OGTs by the two mechanisms: (i) the proton sponge hypothesis suggests that the increased activity of membrane-bound ATPases causes increased influx of H^+^ and Cl^-^ ions which is counteracted by water entry, resulting in swelling and rupture of the vesicles; (ii) the polyplex/polymer mediated escape theory suggests that the cationic polyplex/polymer is further protonated in the acidic environment (also impacted by ATPases activity), leading to enhanced direct interaction with the anionic lipids of the vesicle membrane, causing local destabilization of the membrane integrity and reversible pore formation through which the slow release of the cargo can occur 140,143.

While the precise mechanistic process of endosomal escape still remains unclear, two main mechanisms have been suggested as potential escape routes from the depot endosomes; i) endosomal rupture, also known as the proton-sponge effect, where the endosome irrevocably ruptures releasing its whole cargo into the cytosol (Figure 4); and ii), or polyplex/polymer-mediated enhanced endosomal escape, in which small and reversible breaches in the endosomal lipid bilayer allow small amounts of cargo to escape into the cytosol (Figure 4) 140,142,143. The latter mechanism may potentially explain the prolonged therapeutic effect observed in both experimental and clinical settings 144. However, localized disruption of the endosomal lipid bilayer membrane is a rare and transient event that represents the rate-limiting step in the endosomal escape process 140. In contrast, endosomal or lysosomal rupture can induce non-specific cytotoxicity as it is known to activate the inflammasome 145 that may lead to cell death 146. The acidic environment within endosomes stimulates the endosomal escape of the entrapped cargo. The efficiency of the escape is also affected by several proteins of the endosomal and lysosomal systems 147-150. A better understanding of these mechanisms could offer strategies for enhancing endosomal escape.

It is believed that cationic and hydrophobic delivery vesicles, nanoparticles and OGT conjugates can facilitate the rate-limiting step of endosomal escape. Cationic conjugates may electrostatically associate with the lumen anionic of endosomal bilayer, while hydrophobic conjugates may penetrate the lipid bilayer. Both processes can cause small transient breaches in the membrane that allow gradual release of OGTs 140. Currently, several of these delivery systems are being actively investigated to increase internalization, enhance release and ultimately increased therapeutic efficacy 151,152.

### 4.4 The choice of the targeted sequence within mRNA affects efficacy

Efficient endosomal escape does not automatically guarantee effective target silencing. For therapeutic action to occur, the OGT must access and efficiently bind specific site within mRNA target. Proper target sequence selection requires consideration of several factors, including the identification of the disease-causing gene and its specific transcript, localization of the target mRNA, target sequence accessibility for OGT binding, conservation of the binding site across gene isoforms, possible nucleotide variations in the targeted sequence and unintended non-specific OGT interactions with the human transcriptome.

Conservation of the targeted site across multiple gene isoforms or even between healthy and mutated transcripts is of the utmost importance. The degree and nature of conservation should be evaluated in the context of the disease, the specific targeted gene, and the intended therapeutic strategy. For example, in the development of antiviral therapeutics, suppression of several essential and conserved genes could inhibit viral replication across multiple strains. In 2022, Yi-Chung Chang *et al.* demonstrated that siRNAs targeting conserved regions of vital Sars-Cov-2 genes such as RDRP, spike, and helicase, significantly inhibited multiple Sars-Cov-2 strains, including Delta. Viral replication decreased by 99% *in vitro* and *in vivo* when tested in infected mice receiving prophylactic treatment 153. Similar findings have been reported for OGT-based antiviral against Sars-Cov-2 154-157, influenza 30,155,156,158-160, HBV 161 and others 156,160,162. Notably, antiviral activity can be elicited by targeting not only vRNA but also the host mRNA. For example, a study by Friefrich *et al.* in 2022 showed that an siRNA targeting exon1 of ACE2 mRNA, which serves as the entry receptor for SARS-CoV-2, reduced both ACE2 mRNA and protein levels by up to 90% for at least six days 163. Perhaps the most successful example of proper target selection is the FDA approved RNAi agent Patisiran against hATTR amyloidosis, which targets so highly accessible and conserved region of the TTR gene that all the subsequently approved OGTs target the same exact site.

Target sequences must also be unique to the target gene to avoid unintended side effects. Computational tools 164 such as siDirect 165, DNAzyme builder 166 are available to create safe OGTs with minimal off-target effects. However, in practice, complete specificity is difficult to achieve. An OGT of approximately 20 nucleotides has a high potential of being partially complementary to multiple sites in the human transcriptome. Such off-target interactions can not only cause side effects but severely reduce on-target activity. Nevertheless, off-target effects are not always detrimental. Interestingly, in the same Yi-Chung Chang's study 153, one of their major off-target was CXCL5, a chemokine involved in humoral immunity. The authors hypothesized that this off-target interaction may have contributed to the antiviral effect of their OGT due to the CXCL5role in COVID-19-associated pathogenesis.

RNA secondary and tertiary structures influence hybridization thermodynamics and targeted site accessibility and thus determine OGT efficiency. It is well documented that ssRNA sites, such as loops, are more accessible and show better suppression efficiency when targeted than dsRNA regions such as stems 167,168. For instance, Cas13 gRNAs targeting dsRNA regions of the long non-coding RNA XIST showed low gene silencing), which increased by almost five-fold when single-stranded regions of the same RNA were targeted instead 169. At the same time, RNA binding domains (RBDs) that can be occupied by proteins should be considered during target site selection as RNA binding proteins (RBPs) can inhibit gene silencing via steric hindrance or enhance it via natural RBP interactions such as those observed for Ago/miRNA mediated gene regulation 170. High ribosomal activity can also make the target site more accessible by unfolding its complex structure, facilitating RISC interactions and higher siRNA efficiency 171. RNA secondary structure can be predicted by several tools, such as RNAstructure 172 or icSHAPE 173. However, *in vitro* assays still offer a more accurate assessment by confirming the presence of stable, conserved structural elements, while also revealing many previously unknown structures 174. For instance, Kierzek's group has extensively studied viral RNA structural motifs for OGT design. They identified regions in segment 5 of IAV mRNA that were accessible by ASO and RNAi agents 175,176, and achieved nearly 90% reduction of the viral titer 177 thus demonstrating how knowledge of secondary RNA structures can help in OGT design.

### 4.5 OGTs' design can increase efficiency by protecting from nuclease degradation, achieving affinity/specificity balance and enhancing OGT/protein interactions

To increase the probability of successful target binding, OGT design guidelines recommend avoiding sequences with strong internal secondary structures, maintaining a GC content of 30-50%, avoiding runs of four or more identical nucleotides, and excluding sequences that can target unintended genes due to partial sequence complementarity. Commonly, ~20 nt long OGT/target hybridization sites show the best knockdown efficiency, due to their sufficient affinity for the target under physiological conditions 178-181. Lower affinity can reduce silencing efficacy due to unstable binding. However, the high affinity of the 20 nt OGT may cause non-specific binding to partially complementary fragments of unintended targets. The affinity/specificity dilemma suggests that the higher the affinity is, the lower the specificity of OGTs and vice versa 4,182. The fragile OGT/mRNA complex stability should be fine-tuned by careful sequence design and/or by introducing chemical or structural alterations that can help attain an optimal affinity/specificity balance.

Advances in ON chemistry have improved ASO performance 183, with the phosphorothioate (PS) backbone remaining prevalent for its role in ON trafficking 184 and RNase H recruitment 185. However, PS alone is insufficient to fully protect ASOs from nucleases and can increase cytotoxicity. This has led to the development of alternative backbones like mesylphosphoramidate (MsPA) 186,187. For instance, Patutina *et al.* reported up to 90% target silencing *in vitro* after 72 h post-transfection with 100 nM MsPA ASOs, and up to 95% target silencing *in vivo* compared to approximately 50% silencing by PS-modified ASOs 188. Sugar modifications such as 2'-O-methyl (2'OMe), 2'-methoxyethyl (2'MOe) 189,190 and locked nucleic acids (LNA) 191-193 have enhanced both stability and binding affinity but do not support RNase H activity. To address this problem, the 'gapmer' design was introduced. It combines central PS or native DNA to support RNase H activity with affinity-boosting modifications on the 5' and 3' ends 194. LNA gapmers are among the most effective, allowing fine-tuning of affinity and demonstrating superior efficacy. Shin *et al.* have shown that an LNA gapmer achieved approximately 90% *in vivo* gene silencing in the lungs of mice two days after intratracheal administration, outperforming 2'MOe gapmers that achieved only 60% gene silencing 195. Gapmers combining LNA, cEt, 2'-FANA, and 2'-5 linkages have been utilized for SNP-selective targeting 158,196,197. Nonetheless, excessive ASO/target affinity can be counterproductive by limiting ASO recycling and depleting the ASO pool through off-target binding 198,199, a challenge shared by other nucleic acid systems guided by nucleases or ribozymes 200,201.

siRNA modifications should not affect Ago activity and not alter the agent's conformation, as an RNA-like conformation is essential for RISC assembly and activation. Extensive studies have shown that strategically placed modifications, like alternating 2'OMe and 2'-fluoro (2'F) or PS linkages at the edges of each strand, enhanced siRNA stability without compromising activity 142,202. Hassler *et al.* reported that fully modified siRNA achieves efficient tissue accumulation, RISC loading, and gene silencing, with an IC50 of 0.9 nM versus 3.5 nM of native siRNA 203. The 5'-phosphate of siRNA's guide strand is critical for RISC recognition. Chemical stabilization, such as introducing 5'-E-vinylphosphonate (5'-E-VP) and PS modifications, strengthens the 5'-end against nucleases without impeding RISC interaction, improving IC50 of 81 nM compared to 217 nM for native siRNA against PPIB mRNA, as shown by Haraszti *et al.* 204. 5'-E-VP introduction was also shown to extend the siRNA-based silencing duration for over 30 days in rapidly dividing cells 205. Special attention is given to the seed region of the siRNA guide strand. Fully or partially modified seed regions can alter both affinity and specificity to the intended target either positively, by reducing off-targets, or negatively, by disrupting structural integrity of the region thus inhibiting target binding 206-208. It has been proposed, though, that the seed region should be considered a dual-functional domain and optimized to balance high target affinity and low off-target activity when modified with 2'OMe 209.

The role of the passenger strand must also be considered since it can be mistakenly loaded into the RISC, reducing efficacy and producing off-target effects. Some modifications such as LNA can significantly inhibit RISC activity if introduced in the guide strand. However, they may be useful for minimizing the unintentional passenger strand activity 208. Likewise, bulky phosphoryl guanidine (PG) groups at the 5' end of the passenger strand can be used to reduce off-target effects, leading to approximately 0.5 fold change (FC) in on-target silencing compared to 0.9 FC of non-PG-modified siRNA 210. Since 5' end modification can increase the RISC loading probability of a strand, modifying the 5' end of the guide strand can also help avoid unintended off-targets 211.

DZs and RZs, on the other hand, are better predisposed to address the affinity/specificity dilemma due to their use of two relatively short arms that cooperatively bind targeted RNA. The length of these arms can be adjusted to increase affinity to their target 212, increase specificity against SNVs 76, or be asymmetric to achieve efficiency/specificity balance 213,214. DZs and RZs are governed by a well-characterized enzymatic cycle: 1) target binding, 2) catalytic reaction and 3) product release. Each step is crucial for achieving optimal reaction efficiency, and arm lengths can significantly influence both the initial binding and final release steps. Contrary to the earlier assumptions that high affinity Dz arms inhibit product release, recent findings suggest that longer high affinity arms increase Dz catalytic efficiency by facilitating RNA substrate binding, which is the limiting stage of the catalytic cycle. Very long arms with T_m_ > 65^o^C are not inhibited by the product release stage, as long as the cleavage products self-fold into stable secondary structures 22,182,215,216.

High affinity of Dz to RNA can be achieved by introducing LNA nucleotides in short flanking arms 217-219. However, chemically modifying the catalytic core remains challenging, as even small chemical modifications often inhibit the catalytic activity 17,220,221. This limitation impairs intracellular performance, as the unmodified core is vulnerable to nuclease degradation. To overcome these issues, researchers have explored strategic combinations of chemical modifications. These approaches have achieved up to a two-fold or greater reduction in target gene expression in cell-based studies 219. *In vitro* selected chemically modified variants of Dzs and Rzs have shown promising results achieving more than fourfold increase in Dz activity and a twofold higher Rzs activity compared to unmodified equivalents 222-224.

The CRISPR/Cas13 system obeys several key rules for optimal activity: it targets an ssRNA region, requires perfect matching between gRNA and target sequences especially with the 'seed region' and avoids stable gRNA secondary structure. It has also been shown that fusion of gRNA with a nuclear localization signal (NLS) can increases efficacy of Cas13 systems 225. Although the CRISPR/Cas13 system modifications are not yet thoroughly studied, some data suggests that 2'OMe and PS modifications placed at the 3' end of the gRNA can increase its transient target silencing in human T-cells from 40-45% to 60-65% 226.

Stereopure mixtures of OGTs have been proved to outperform the respective stereorandom counterparts 227,228. Researchers use specific chemistries to tightly control the chiral configuration of the modified backbones. For example, chimeric PS/PN (phosphoryl guanidine) containing backbones developed by Kandasamy *et al.* 229, displayed enhanced activity both *in vitro* and *in vivo* with better pharmacological properties than stereorandom PS modified splice switching ONs. This improvement enabled a twofold reduction in dosage during in vivo testing 229. Chiral chemical modifications have also been shown to affect siRNA agents 230,231, although other OGTs have yet to be studied in this context. However, this approach doesn't come without challenges-nuclease stability remains a significant concern and one of the major obstacles to its broader application 232,233.

Design can also affect the immunogenicity of OGTs. OGTs rich in guanosine (G) and uridine (U), and motifs such as CpG, GU and AU, are recognized as viral RNA mimics and can activate toll-like receptors 3, 7 and 8 (TLR3, TLR7 and TLR8 respectively) 234. Pollak *et al.* have shown that native and modified OGTs, especially PS-modified, induce innate immune activation, by interacting with several extracellular proteins and TLR9 235. Such interactions ultimately lead to cytokine and interferon production which can interfere with the cellular uptake, trafficking, and processing of the oligonucleotides, thereby reducing their ability to engage target mRNA effectively. It can also lead to the activation of the complement system and opsonization of OGTs, which promotes their clearance by phagocytes, lowers their bioavailability and half-life in circulation and tissues, thus diminishing their effective concentration at the target site. To mitigate these effects design strategies such as reducing the PS content and strategic placement of 2'OMe have been proposed 235,236. While well-documented adverse effects such as injection site reactions, fever, chills, and systemic inflammation can complicate clinical efficacy, many studies tend to underemphasize the immunogenic effect of OGTs.

## 5. Theoretical vs real world efficacy

The reported efficacy of OGTs in modern literature often falls short of theoretical expectations. The expected efficacy of OGTs is typically derived from computational and empirical models designed to predict how effectively OGTs will silence target genes. Such theoretical indexes include i) the Secondary Structure Score (Sscore), which estimates the strength of local mRNA secondary structures at the OGT target site; ii) the Duplex Score (Dscore), which estimates the stability of the OGT:mRNA duplex formation; and iii) the Competition Score (Cscore), which represents the difference between Dscore and Sscore 237. These indices are incorporated into major designing tools such as SciTools suite by IDT 238 and Ufold 239. Well-designed OGTs, with the help of these tools, are expected to achieve over 70% knockdown of the mRNA target under optimal conditions.

However, a 2016 study by Munkacsy *et al.*, revealed a significant gap between theory and practice 240. After evaluating 1643 samples across 429 experiments published in 207 siRNA studies, they found that 70% knockdown — was achieved in only 10 experiments (2.3%); 50% in 166 experiments (38.7%), and 30% in 79 experiments (18.5%) of the cases. Surprisingly, the unexpected upregulation was observed in 22 experiments (5.1%). The study concluded that the choice of the cell line and the validation method had the most impact on silencing efficiency. It also highlighted issues such as the improper use of controls and variability in experimental conditions, which contribute to inconsistencies and raise questions about reliability of the results. According to a recent study of Davis *et al.*, target-specific features, such as exon presence, ribosome occupancy and so on, may likely be responsible for the low endogenous efficacy of siRNA 171. This highlights the need for native expression assay inclusion in OGT development studies, that is often replaced by reporter-based expression assays.

Considering only ~1% of the internalized OGTs escape into the cytosol, the low efficiency of most OGTs might not look as disappointing as it initially seems. The inconsistency of theoretical prediction with experimental findings indicate that intrinsic aspects of OGT-assisted gene silencing are yet to be understood.

## 6. Concluding remarks

Although extensive research has led to considerable advancements, significant challenges still hinder the full potential of OGTs in medicine. The low efficiency of OGTs remains a primary barrier to their successful translation and widespread adoption in medical practice. Tissue specific delivery, efficient internalization and endosomal escape, are the major factors affecting OGT efficacy. Despite the growing use of cationic and lipid-based delivery systems, the percentage of OGTs that escape from endosomes is disappointingly low, estimated at less than ~1%. This result suggests that poor endosomal escape is a key factor limiting OGT efficiency and their overall therapeutic performance.

While experimental studies have characterized siRNA as the most potent among OGTs, evidence suggests that only 2.3% of siRNAs reach the theoretically predicted 70% target downregulation, while approximately 40% of the siRNAs fail to achieve 50% suppression level. These results highlight the inadequacy in siRNA design and the challenges associated with achieving consistent and effective gene silencing across different studies and experimental conditions. Inconsistent methodologies, and suboptimal controls further obscure accurate assessments of OGT performance, challenging their reliability as a therapeutic tool and halting the advancement of the field. Additionally, the lack of standardized reporting, such as varying concentration units (molarity vs. mass), different assay methodologies, and endpoint variability, including short-term vs. long-term effects, further impedes result validation and can confuse the reader ultimately eroding trust in the findings. Standardizing assays and reporting are ongoing and adopting existing (e.g. MIQE for PCR data), or developing new guidelines will help in comparative data analysis.

These broader challenges are also reflected by the performance of approved OGTs in clinic that often only marginally improve patient conditions. The limited clinical success of the approved OGTs is likely due to their suboptimal performance when compared with pre-existent therapeutics or a lack of alternative treatment options. Notably, ASOs, generally less efficient than the most potent siRNA, have higher success rates in FDA and EMA approval. Moreover, statistical data shows that more ASOs have entered clinical trials and moved to more advanced phases, than siRNA and miRs combined. Likely, challenges in siRNA design can be attributed to the greater complexity in designing and modifying them without reducing activity compared to ASO. On the other hand, miRs face challenges due to the inherently low selectivity making them less studied in both experimental and clinical settings. The Dz agents have not been successful in clinical trials due to the incorrect design of RNA binding arms, having too low affinity to targeted RNA, and the difficulty in chemical protection of their catalytic cores against nuclease degradation. CRISPR/Cas13 remains relatively new and not sufficiently studied tool that might be limited by greater challenges in delivery in comparison with ASO and siRNA, low specificity and collateral RNase activity. Notably the recent approval of Imetelstat, a first-in-class oligonucleotide agent that acts as a telomerase inhibitor, offers hope by restoring normal haematopoiesis for low to intermediate risk patients with anaemia.

Ultimately, while OGTs still face considerable hurdles, we are now at the stage when incremental improvements in delivery, design, or validation of OGT efficacy could unlock immense potential, revolutionizing gene therapy.

## Figures and Tables

**Figure 2:**
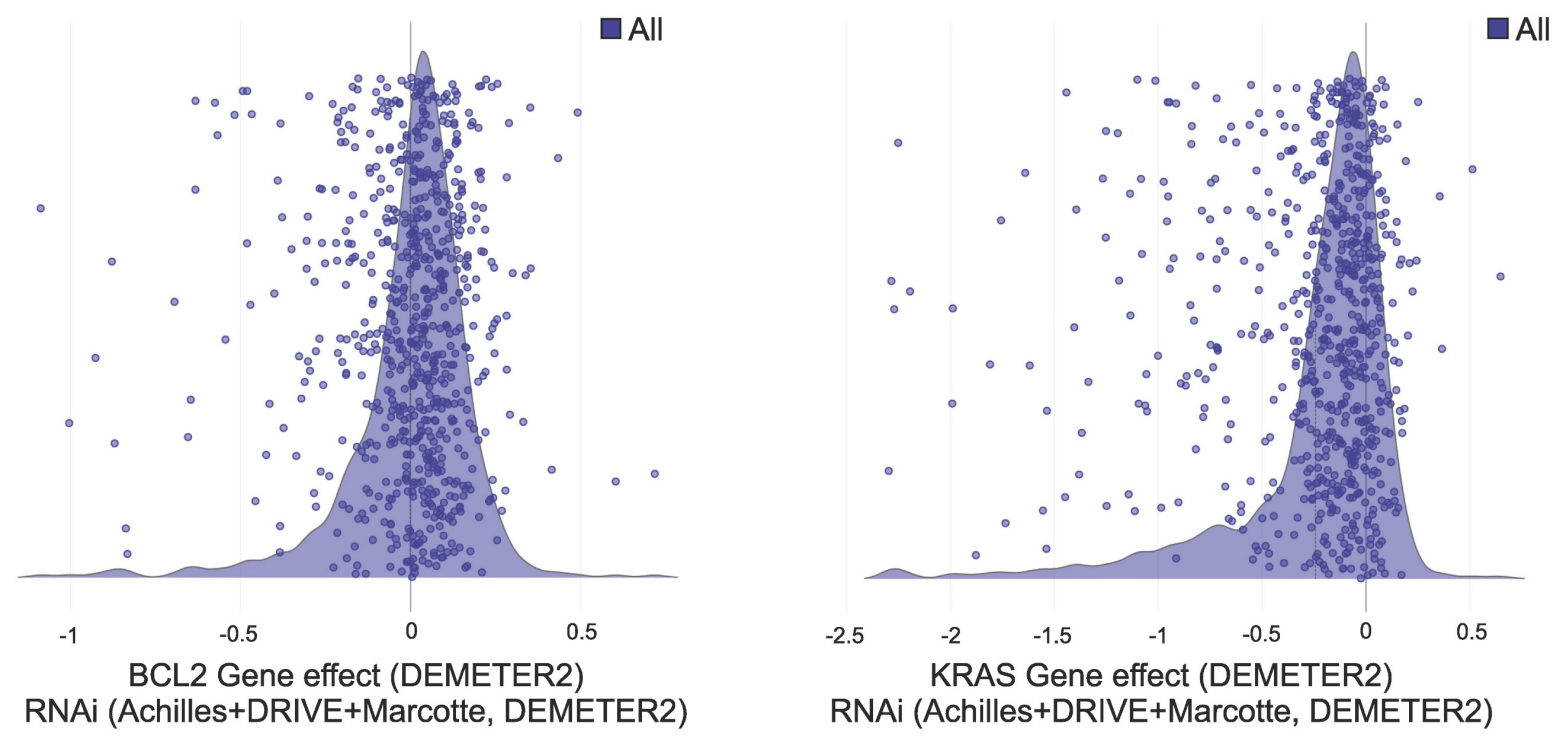
Graphs representing the gene effect (RNAi, Achilles+DRIVE+Marcotte, DEMETER2) of A) BCL2 and B) KRAS genes. Each purple dot on the graph represents a cell line on which the gene effect of the respective gene was tested by cell depletion assay. In the DEMETER2 scoring system, lower scores indicate higher gene essentiality; a score of 0 suggests the gene is non-essential in that cell line; a score of -1 approximates the median score for pan-essential genes, reflecting high essentiality. For both BCL2 and KRAS, most cell lines fall within the gene effect range of [-0.5, 0.5], suggesting low essentiality, while only a limited number of cell lines exhibit scores approaching or exceeding -1.

**Table 1:** FDA/EMA approved OGT formulations.

Drug Name	Agent Type	Approval	Target Disease	Target Gene	Delivery Vehicle	Efficacy Data	Dosing Regimen
Fomivirsen (Vitravene)	ASO	FDA/EMA: 1998/1999 (Discontinued in 2004/2002)	Cytomegalovirus retinitis	CMV IE2 mRNA	N/A	Significant reduction in CMV replication in the eye	330 µg once weekly for up to 3 weeks / IT injection
Mipomersen (Kynamro)	ASO	FDA/EMA: 2013 (Discontinued in 2019)	Homozygous familial hypercholesterolemia (HoFH)	ApoB	N/A	Reduced LDL-C levels by approximately 25%	200 mg once weekly /SC injection
Nusinersen (Spinraza)	ASO	FDA/EMA: 2016	Spinal muscular atrophy (SMA)	SMN2	N/A	Improved motor function in SMA patients; achieved motor milestones in ~40% of treated patients	Initial: Loading dose of 12 mg on days 0, 14, and 28; Maintenance: every four months thereafter / IT injection
Milasen	ASO	compassionate use - 2018	Batten disease	CLN7	N/A	Improved motor function and cognitive abilities in case studies	42 mg once every 3 months / ITH
Inotersen (Tegsedi)	ASO	FDA/EMA: 2018	Hereditary transthyretin amyloidosis (hATTR)	TTR	N/A	Significant reduction in serum TTR levels (~80%)	284 mg weekly for first three weeks / SC injection
Eteplirsen (Exondys 51)	ASO	FDA: 2016	Duchenne muscular dystrophy (DMD)	DMD	N/A	Increased dystrophin levels by ~0.93% of normal after 180 weeks; improved motor function in some patients	30 mg/kg once weekly / IV infusion
Golodirsen (Vyondys 53)	ASO	FDA: 2019	Duchenne muscular dystrophy (DMD)	DMD	N/A	Increased dystrophin production in muscle tissue by ~60%	30 mg/kg once weekly / IV infusion
Viltolarsen (Viltepso)	ASO	FDA: 2020	Duchenne muscular dystrophy (DMD)	DMD	N/A	Increased dystrophin production in muscle tissue by ~60%	80 mg/kg once weekly / IV infusion
Casimersen (Amondys 45)	ASO	FDA: 2021	Duchenne muscular dystrophy (DMD)	DMD	N/A	Increased dystrophin production in muscle tissue by ~60%	30 mg/kg once weekly / IV infusion
Tofersen (Qalsody)	ASO	FDA: 2023	Amyotrophic lateral sclerosis (ALS)	SOD1	N/A	Significant reduction in SOD1 levels; improved clinical outcomes in treated patients	Initial: Loading dose of 100 mg; Maintenance: every four weeks thereafter / IT injection
Volanesorsen (Waylivra)	ASO	EMA: 2019	Familial chylomicronemia syndrome	ApoC-III	N/A	Reduces triglyceride levels significantly	Initial dose followed by maintenance dose every week
Patisiran (Onpattro)	siRNA	FDA/EMA: 2018	Hereditary transthyretin amyloidosis (hATTR) polyneuropathy	TTR	Lipid nanoparticles (LNPs)	80% reduction in serum TTR levels after 18 months	Initial: 0.3 mg/kg IV every 3 weeks (<100 kg); Maintenance: 30 mg IV every 3 weeks (≥100 kg) / IV infusion
Givosiran (Givlaari)	siRNA	FDA/EMA: 2019/2020	Acute hepatic porphyria (AHP)	ALAS1	GalNAc-conjugated siRNA	Significant reduction in ALA levels during acute attacks	2.5 mg/kg once monthly / SC injection
Lumasiran (Oxlumo)	siRNA	FDA/EMA: 2020	Primary hyperoxaluria type 1 (PH1)	HAO1	GalNAc-conjugated siRNA	Significant reduction in urinary oxalate excretion by ~60% at 6 months	Initial: 3 mg/kg once monthly; Maintenance: 1 mg/kg SC once monthly / SC injection
Inclisiran (Leqvio)	siRNA	FDA/EMA: 2021/2020	Heterozygous familial hypercholesterolemia and atherosclerotic cardiovascular disease	PCSK9	GalNAc-conjugated siRNA	Significant reduction in LDL-C levels by ~50% at 6 months	Initial: 284 mg at day 1 and day 90; Maintenance: every 6 months / SC injection
Vutrisiran (AMVUTTRA)	siRNA	FDA/EMA: 2022	Hereditary transthyretin amyloidosis (hATTR) cardiomyopathy	TTR	GalNAc-conjugated siRNA	Sustained reduction in serum TTR levels over dosing interval (up to ~80%)	Loading: 25 mg once monthly for three months; Maintenance: every three months / SC injection
Nedosiran (Rivfloza)	siRNA	FDA/EMA: 2023	Primary hyperoxaluria type 1 (PH1)	GPD1L	GalXC™ RNAi platform	Significant reduction in urinary oxalate excretion by ~70% at month 6	Initial: 3 mg/kg once every month; Maintenance: every three months / SC injection

**Table 2:** Essentiality of several cancer related genes that are commonly used as targets in cancer gene silencing research. The overall gene effect of each gene (DEMETER2 score) was calculated as the mean of its gene effect on each distinct cancel cell line included in the DepMap Portal database (as shown in Figure 2).

Gene	Gene Effect RNAi (Achilles+DRIVE+Marcotte, DEMETER2)	Druggable Structure
KRAS	-0.06	yes
BCL-2	0.04	yes
MYC	-0.5	yes
BMI1	-0.03	yes
EGFR	0.05	yes
BRCA1	-0.1	yes
CXCL14	-0.1	no
YAP	0.03	no
IDO1	0.1	yes
TP53	-0.1	yes
mTOR	-0.5	yes
STAT3	-0.1	yes
VEGFA	-0.1	yes

## Abbreviations

ALS: amyotrophic lateral sclerosis
ALN-HBV: RNAi agent targeting hepatitis B virus
ApoB: apolipoprotein B
ApoC-III: apolipoprotein C-III
ASO: antisense oligonucleotides
ASGR: asialoglycoprotein receptor
AHP: acute hepatic porphyria
Bcl-2: B-cell lymphoma 2
BP1002: Bcl-2-targeting antisense oligonucleotide
cEt: constrained ethyl
CMV: cytomegalovirus
CPP: cell-penetrating peptide
CRISPR: clustered regularly interspaced short palindromic repeats
crRNA: CRISPR RNA
CXCL5: chemokine ligand 5
DMD: Duchenne muscular dystrophy
DNA: deoxyribonucleic acid
Dzs: DNAzymes
EMA: European Medicines Agency
FDA: United States Food and Drug Administration
FCS: familial chylomicronemia syndrome
GalNAc: N-Acetylgalactosamine
GPD1L: glycerol-3-phosphate dehydrogenase 1-like protein
gRNA: guide RNA
HBsAg: hepatitis B surface antigen
HBV: hepatitis B virus
HDL: high-density lipoprotein
HIV: human immunodeficiency virus
hATTR: hereditary transthyretin-mediated amyloidosis
IC50: half maximal inhibitory concentration
IE2: immediate-early protein 2
IT: intrathecal
IV: intravenous
KRAS: Kirsten rat sarcoma viral oncogene homolog
LDL: low-density lipoprotein
LDL-C: low-density lipoprotein cholesterol
LNA: locked nucleic acid
LNP: lipid nanoparticle
miR: microRNA
miRNA: microRNA
MOe: 2'-methoxyethyl
MsPA: mesylphosphoramidate
mRNA: messenger RNA
N/P: nitrogen to phosphate ratio
NLS: nuclear localization signal
OGT: oligonucleotide-based gene therapy/therapeutics
ON: oligonucleotide
ORF: open reading frame
PCSK9: proprotein convertase subtilisin/kexin type 9
PG: phosphoryl guanidine
PH1: primary hyperoxaluria type 1
PPIB: peptidylprolyl isomerase B
PS: phosphorothioate
qPCR: quantitative polymerase chain reaction
RISC: RNA-induced silencing complex
RNA: ribonucleic acid
RNAi: RNA interference
RNP: ribonucleoprotein
Rz: ribozyme
Rzs: RNAzymes
RSV: respiratory syncytial virus
SARS-CoV-2: severe acute respiratory syndrome coronavirus 2
SC: subcutaneous
SCORE: Secondary structure, duplex, and competition score index
siRNA: small interfering RNA
SMN2: survival motor neuron 2
SNV: single nucleotide variant
SOD1: superoxide dismutase 1
SR-B1: scavenger receptor class B member 1
ssDNA: single-stranded DNA
ssRNA: single-stranded RNA
TGF: transforming growth factor
Tf-PEI: transferrin-polyethyleneimine
Tm: melting temperature
TP53: tumor protein p53
TTR: transthyretin
UTR: untranslated region
VEGF: vascular endothelial growth factor
vRNA: viral RNA
ZFN: zinc finger nuclease
